# Straw mulch improves soil carbon and nitrogen cycle by mediating microbial community structure and function in the maize field

**DOI:** 10.3389/fmicb.2023.1217966

**Published:** 2023-07-18

**Authors:** Bangyan Liu, Yisha Dai, Xin Cheng, Xian He, Qicheng Bei, Yifan Wang, Yuling Zhou, Bo Zhu, Kangping Zhang, Xiaoqin Tian, Meichun Duan, Xiaoyu Xie, Longchang Wang

**Affiliations:** ^1^College of Agronomy and Biotechnology, Southwest University, Chongqing, China; ^2^Engineering Research Center of South Upland Agriculture, Ministry of Education, Southwest University, Chongqing, China; ^3^Department of Soil Ecology, Helmholtz Centre for Environmental Research - UFZ, Halle, Germany

**Keywords:** straw mulch, microbial community structure, carbon cycle, nitrogen cycle, carbon metabolism

## Abstract

This study was conducted to investigate the capability of the microbial community characteristics and soil variables to promote carbon and nitrogen cycles in maize fields under straw mulch. We covered the surface soil of the maize field with different amounts of wheat straw (0 kg/ha, 2,250 kg/ha, and 4,500 kg/ha) and used 16S rRNA and ITS sequencing, Biology ECO-plate, traditional enzymology, TOC analyzer, and HPLC to measure bacterial and fungal community composition and functions, characteristics of microbial carbon source metabolism, carbon and nitrogen fraction, enzyme activity, and organic acid content in the maize rhizosphere and non-rhizosphere. The results indicated that short-term straw mulch insignificantly affected the alpha diversity of bacterial and fungal communities whereas significantly influenced their beta diversity. The results of functional prediction revealed that straw mulch considerably boosted the relative abundances of bacteria belonging to chemoheterotrophy, aerobic chemoheterotrophy, ureolysis, and nitrogen fixation and inhibited fermentation and nitrate reduction in maize rhizosphere soil. These processes primarily drove the C and N cycles in soil. Straw mulch also improved fungal saprotrophs by raising the proportion of *Chaetomiaceae* and *Chaetosphaeriaceae*. The Biology ECO-plate results illustrated that straw mulch weakened the metabolism capacity of microbial labile carbon resources. As a result, the labile C and N fractions were raised under straw mulch. Our results also showed that straw mulch primarily regulated the microbial community structure in rhizosphere soil by significantly decreasing *Firmicutes* and *Ascomycota* relative abundance while increasing *Basidiomycota*. The fungal community structure is more than bacterial for affecting soil microbial biomass carbon, readily oxidizable organic carbon, dissolved organic carbon, available nitrogen, ammonium, and nitrate directly and indirectly through malic acid content and cellulase, protease, and amylase activity. Overall, our findings imply that straw mulch might influence the bacterial and fungal community structures, thereby boosting the production of labile C and N components and accelerating the C and N cycle in maize fields.

## 1. Introduction

The process of agricultural production generally produces a large number of straw residues such as corn stover/cob (1,661 million tons), rice straw (975 million tons), and wheat straw (529 million tons) every year in the world (Tan et al., 2021). Otherwise, the majority of straw is directly discarded or burned, leading to biomass resource waste and environmental pollution (Huang et al., 2021). Straw burning, in particular, seriously influences global climate change and public health by increasing fine particulate matter and trace gas (e.g., CO_2_, CH_4_, N_2_O, SO_2_) emissions (Chen et al., 2019; Ren et al., 2019; Huang et al., 2021). Hence, the strategies of straw residue utilization have received a lot of attention from researchers in recent decades, and it has been found that straw returning to the field such as mulching is the most economical and environmental measure for straw residue deposal (Li et al., 2018; Cong et al., 2020).

Straw mulch, as one of the conservation agricultural management and cultivation practice, provides a large of energy and food for the activities of soil microorganisms, soil animals, and crops during the degradation process, realizing the organic cycle of bioenergy and playing a vital role in ecologically sustainable agriculture (Liu et al., 2017; Guo et al., 2018; Sun et al., 2018). Simultaneously, straw degradation involves a series of complex microorganism processes (Mei et al., 2020). For instance, white rot fungi can degrade the lignin of straw and convert complex polysaccharides into simple sugars, which can be used as carbon resources by bacterial communities or plant roots (Sharma and Arora, 2015; López-Mondéjar et al., 2016; Maarastawi et al., 2019). In general, microorganisms are involved in diverse soil biogeochemical processes, such as litter decomposition, nutrient mineralization, and soil carbon or nitrogen cycles, through the detrital food chain by interacting with soil fauna (Bardgett and Van Der Putten, 2014; Grandy et al., 2016; Kou et al., 2020). For instance, bacterial communities (e.g., *Acidobacteria, Proteobacteria*, and *Actinobacteria*) primarily mediate the degradation of easily decomposed organic matter such as fat and sugar in the first stage of straw degradation, whereas fungal communities (e.g., *Basidiomycota* and *Mortierellomycota*) mainly degrade cellulose, hemicellulose, lignin, and other refractory substances of straw at the latter stage (Paterson et al., 2008; Marschner et al., 2011; Weng et al., 2021). Therefore, soil microbes, as the vital hinge for energy and matter exchange between the biosphere and abiotic sphere in the soil ecosystem, play an important role in the transformation of straw organic carbon and nitrogen into the soil during the process of straw decomposition (Zhang et al., 2016; Cong et al., 2020; Lu et al., 2020). Hence, researching the series of soil biochemical processes driven by the change of microbial communities with straw mulch application is incredibly beneficial for further understanding the material cycle in the agricultural soil ecosystem.

The straw contains the majority of organic components such as lignin, cellulose, starches, lipids, and proteins that can regulate soil organic carbon or nitrogen storage and compensate for the loss of native soil C and N in agroecosystems by returning (Liu et al., 2014; Cao et al., 2018; Hou et al., 2020; Zheng et al., 2020). Sun et al. (2013) studied that straw return directly enhanced soil organic carbon (SOC) storage in the 0–20 cm soil layer. Hao et al. (2022) illustrated that mineral fertilizers plus straw return significantly increased the contents of soil readily oxidizable organic carbon (ROC) and particulate organic carbon (POC) and their proportion in SOC. Similarly, Cong et al. (2020) found different types of straw return increased the soil-dissolved organic carbon (DOC) and microbial biomass carbon (MBC) contents by regulating C-related microbial abundance. Researchers also agreed that wheat straw could minimize soil N leaching and runoff potential while boosting N immobilization and indicated that N fixation is primarily caused by fungi when the straw decomposes (Zavalloni et al., 2011; Cao et al., 2018). Furthermore, Xia et al. (2018) supposed that the C and N biogeochemical cycles are closely coupled and that straw return enhances soil C storage while also affecting soil reactive N losses. Therefore, studying the soil C and N fractions mediated by microbial communities changing with a straw mulch will benefit further understanding the biogeochemical cycling of the organic matter resulting from straw.

Soil enzymes, including extracellular and intracellular enzymes, play a major role in the biochemical cycle of organic matter in the soil system, and their activities are closely linked to soil physical characteristics, SOM, and microbial biomass and activity (Karaca et al., 2011). For example, cellulase and urease play key roles in carbon and nitrogen cycling in the soil and are excellent indicators of soil biological change because they react swiftly to soil management (Wang et al., 2012; Utobo and Tewari, 2015). According to Akhtar et al. (2018), the soil enzyme activities that are connected to SOC and accessible N concentrations were dramatically reduced by the straw mulch. Some researchers documented that the microorganisms degraded the cellulose and lignin in straw into various carbohydrates or other smaller organic components by secreting extracellular enzymes (Salvachúa et al., 2013; Shen et al., 2020). In addition, soil organic acids, originating from plant root exudates, microorganism excretions, and organic matter decomposition, play crucial roles in the carbon cycle, metal detoxification, and physiochemical processes of mineralization and solubilization of poorly available and complex minerals in soil (Adeleke et al., 2017). Hence, by comprehensively analyzing the soil enzyme activity and organic acid content interactions with the microbial community structures and functions changing with straw mulch, we can deeply explore the soil biogeochemical succession process mediated by straw mulch.

Recent studies on straw mulch patterns regulating microbial communities and SOC fractions have increased (Wang et al., 2018; Cong et al., 2020; Jin et al., 2020), though few have examined the microbial community characteristics stimulated by straw mulching to regulate soil carbon and nitrogen fractions, organic acids, and soil enzyme activities. Thereby, this research was conducted by using wheat straw mulch patterns with varying amounts (0 kg/ha, 2,250 kg/ha, and 4,500 kg/ha), applying 16S DNA and ITS amplicon sequencing to identify the bacterial and fungal community composition and predict their functions, respectively. Additionally, the Biological ECO-plate technology was applied to test the microbial carbon metabolism ratio (Guo et al., 2020; Liu et al., 2022). Simultaneously, the measurements of soil organic acids, enzyme activity, and C and N fractions were made using HPLC (high-performance liquid chromatography), classic enzymology methods, and a TOC analyzer. The aims of this study were (i) to investigate how the soil microbial community diversity, composition, and functions changed with increasing straw mulch amount in maize rhizosphere and non-rhizosphere soil; (ii) to assess the content of soil C and N fractions mediated by microbial communities changing with straw mulch; and (iii) to study the interaction mechanism among soil organic acids, enzymes activity, soil C and N fractions, and microbial communities regulated by straw mulch.

## 2. Materials and methods

### 2.1. The study design and samples collection

This study was performed on the experiment farm at Southwest University, Chongqing, China (29°81′N, 106°41′E; elevation 244 m). The soil type was classified as purple soil (dystric regosols) on dry land (Liu et al., 2022). Before the study, the soil pH was 6.0, the soil moisture content was 23.73%, the soil organic matter (SOM) content was 18.62 g.kg^−1^, and the soil total and available nitrogen contents were 1.065 g·kg^−1^ and 99.35 mg^.^kg^−1^, respectively.

The cultivation pattern was wheat–maize–soybean rotation–intercropping, with the following details: each plot area was 28.8 m^2^ (3.6 m × 8 m) and divided into eight strips (3.6 m × 1 m), with two crops intercropping in separate strips of the same plot, and each crop was planted four strips per plot. Wheat–maize intercropped from 20 April to 9 May 2019, following which soybean was cycled in wheat strips, and maize–soybean intercropped from 11 May to 14 August 2019. Maize seedlings were nurtured in a 25°C incubator (15,000 lx light intensity, 70% humidity) for 7 days before being planted in the field on March 20, and each strip grew two rows with ten seedlings per row. Wheat was grown from 2 November 2018 to 9 May 2019, with soybean planted in wheat strips on May 11. Three mulch amounts of wheat straw were designed, i.e., CK (0 kg·hm^−2^ straw mulch amount), S1 (2,250 kg·hm^−2^ straw mulch amount), and S2 (4,500 kg·hm^−2^ straw mulch amount). The wheat straw was cut into ~20 cm long pieces and evenly covered on the maize field on May 23, using a randomized block design with three repetitions for a total of nine plots. The basic fertilizers were applied with a compound fertilizer of 750 kg·hm^−2^ (including N 15%, P_2_O_5_ 15%, and K_2_O 15%) and urea of 150 kg·hm^−2^.

During the pustulation stage (26 June 2019), samples of maize rhizosphere and non-rhizosphere soils were obtained. With five plants per plot, by shaking the maize root to remove loose dirt, only soil attached to the root surface at 0–2 mm was preserved as rhizosphere soil samples. To sample the non-rhizosphere soil, a 0–15 cm soil layer was dug between the maize rows. Impurities were removed from the soils by passing the sample through a 2 mm mesh sieve. A portion of each soil sample was stored at −80°C for sequencing, another portion was stored at 4°C and used to determine the microbial carbon source metabolism and MBC, and the rest portion of the soil samples was air-dried for measuring the physical and chemical properties.

### 2.2. DNA extraction, PCR amplification, sequencing, and data processing

Soil DNA was extracted by using the Fast DNA^®^ SPIN Kit for Soil (MP Biomedicals, California, USA). The extracted DNA quality was measured by using a NanoDrop 2000 UV-Vis spectrophotometer (Thermo Scientific, Wilmington, USA; OD260/280 = 1.86–1.93, and the total DNA concentration range was 29.3–54.0 ng·μl^−1^). The hypervariable regions of bacterial V3–V4 and fungal ITS1 were amplified by using a thermocycler PCR system (GeneAmp^®^ 9700, ABI, USA). The amplified primers of bacteria and fungi were 338F (5′-ACTCCTACGGGAGGCAGCAG-3′) and 806R (5′-GGACTACHVGGGTWTCTAAT-3′) (Xu et al., 2016), and ITS1F (5′-CTTGGTCATTTAGAGGAAGTAA-3′) and ITS2R (5′-GCTGCGTTCTTCATCGATGC-3′) (Adams et al., 2013), respectively. The PCR reaction mixture was as follows: 0.4 μl of FastPfu polymerase (TransGen AP221-02), 4 μl of 5 × FastPfu Buffer, 2 μl of 2.5 mM dNTPs, 0.8 μl of forwarding primer (5 μM), 0.8 μl of reverse primer (5 μM), 0.2 μl of BSA, 10 ng of template DNA, and added ddH_2_O to 20 μl. The PCR amplification conditions were pre-denaturation at 95°C for 3 min, then denaturation at 95°C for 30 s, annealing at 55°C for 30 s, and elongation at 72°C for 45 s as a cycle (bacteria and fungi performed 28 and 36 cycles, respectively), and extension at 72°C for 10 min (PCR, ABI GeneAmp^®^ type 9700). AxyPrep DNA Gel Extraction Kit (Axygen Biosciences, Union City, CA, USA) was used to purify PCR products, and QuantiFluorTM-ST (Promega, USA) was used to quantify DNA.

Illumina's MiSeq PE300 platform was used for amplicon sequencing (Majorbio Biopharmaceutical Technology Co. LTD, Shanghai, China). The Trimmomatic was used to control the quality of original sequence fastq files, and then, the forward and reverse sequences were merged by Flash software (https://ccb.jhu.edu/software/FLASH/index.shtml) in the Mothur platform (Magoč and Salzberg, 2011). The UPARSE software (version 7.1 http://drive5.com/uparse/) was used to cluster the operational taxonomic units (OTUs) at the 97% sequence similarity level, where the singleton and chimera sequences were discarded. RDP Classifier (http://rdp.cme.msu.edu/) was performed to analyze the taxonomy of each 16S DNA and ITS sequence. In total, we obtained 443,448 bacterial sequences and 662,382 fungi sequences from 18 soil samples. Using a 97% similarity cutoff, we further clustered those sequences into 6,095 OTUs for bacteria (Silva: Release138 http://www.arb-silva.de) and 3,168 OTUs for fungi (Unite: Release 8.0 http://unite.ut.ee/index.php), respectively.

### 2.3. The biology ECO-plate experiment

The Biology ECO-plate (Biology, Hayward, CA), including 31 of the most useful labile carbon substrates, could express the carbon source metabolism characteristics of microbial communities (Guo et al., 2020). The incubation and calculating procedures were detailed in our previous study (Liu et al., 2022). The average well-color development (AWCD) was used to calculate the microbial carbon source usage in each microplate as follows: AWCD = ∑(OD_i_ – *R*)/31, where OD_i_ represents the absorbance value of each hole except the control hole, and *R* represents the control hole absorbance value.

### 2.4. The determination of soil physicochemical factors

The soil MBC was measured by using a chloroform fumigation-extraction method (Vance et al., 1987). The soil POC and ROC were determined by sodium hexametaphosphate dispersion and the KMnO_4_ oxidation method, respectively (Li et al., 2015; Hao et al., 2022). Soil total organic carbon (TOC), DOC, and dissolved organic nitrogen (DON) contents were determined using a TOC analyzer (TOC-L; Shimadzu, Kyoto, Japan) (Moon et al., 2019). The soil ammonium nitrogen (AMN) and nitrate nitrogen (NN) contents were tested by the MgO diffusion method and ultraviolet spectrophotometry, respectively (Holmes et al., 1998). The contents of soil total nitrogen (TN) and available nitrogen (AN) were determined by the Kjeldahl method and alkaline hydrolysis diffusion method, respectively (Athalye-Jape et al., 2020; Li et al., 2021).

To measure cellulase activity, 10 g of the soils were incubated with 20 ml of 1% carboxymethyl cellulose solution, 5.0 ml of phosphate buffer (pH = 5.5), and 1.5 ml of methylbenzene for 72 h at 37°C. The released glucose was estimated by the 3, 5-dinitro salicylic acid colorimetry (DNS) method and was expressed in terms of milligrams of Glucose Equivalents per kg of soil per 72 h (mg/kg) (Guan et al., 1986). Similarly, the DNS was used to measure amylase and sucrase activity, which was quantified in milligrams of Glucose Equivalents per gram of soil every 24 h (mg/g) (Guan et al., 1986). The potassium permanganate titration technique was used to assess catalase activity, which was represented in milliliters of KMnO_4_ reduction per gram of soil per hour (ml/g) (Guan et al., 1986). The ninhydrin colorimetry technique was used to assess protease activity, which was expressed in milligrams of NH_2_-N per gram of soil every 24 h (mg/g) (Guan et al., 1986). Urease activity was measured using the sodium phenolate-sodium hypochlorite colorimetry technique, and it was represented in milligrams of NH_3_-N per gram of soil per 24 h (mg/g) (Guan et al., 1986).

Soil organic acids were extracted by a 0.1% H_3_PO_4_ aqueous solution. In brief, 5 g wet soil was dissolved in 10 ml 1% H_3_PO_4_ aqueous solution by shaking for 1 min at room temperature, then 5,000 r/min centrifuged 5 min. The suspension was filtered through 0.45 μm filter membranes for HPLC (Shimadzu prominence UFLC, LC 20 AD, Kyoto, Japan. C18 column: Shim-pack GIST, 4.6 × 250 mm, 5 μm) analysis. Mobile phases A and B were 0.1% H_3_PO_4_ deionized water and acetonitrile with a volume ratio of 98:2, respectively. The detecting ultraviolet wavelength was 210 nm, and the flow rate was maintained at 1 ml/ min. The injection volume was set at 20 μl, and the column temperature was kept constant at 25°C. Finally, by comparing retention durations and areas with pure standards, the organic acids were identified and quantified. Oxalic acid (OA), citric acid (CA), malic acid (MIA), malonic acid (MOA), and succinic acid (SA) were all found in our investigation, but lactic acid, propionic acid, acetic acid, and tartaric acid were not.

### 2.5. Date analysis

The alpha diversity and richness of the microbial community were calculated through the Tukey test of ANOVA analysis to compare the differences within samples, including the indexes of Chao 1, Shannon, Simpson, and others. Based on the Bray–Curtis distance matrix, the non-metric multidimensional scaling (NMDS) and Adonis analysis were generated by the R package of “vegan” to evaluate the microbial community structure variation (Oksanen et al., 2016). The microbial relative abundance was calculated based on the OTU tables, and the R package of “pheatmap” was used to build the heatmap of the microbial community relative abundance and carbon sources metabolism (Kolde, 2019). In addition, the volcano plot of enrichment and depletion OTUs was drawn using the R package “ggplot2” based on the OTUs computation results of the R package of “DESeq2” (Love et al., 2014; Wickham, 2016; Li et al., 2017). We employed the FAPROTAX database platform (http://mem.rcees.ac.cn:8080/) to predict functions of bacterial biogeochemical cycles, such as carbon, nitrogen, and sulfur cycle, as well as other ecological functions (Zhang et al., 2019; Yu et al., 2021). The FUNGuild database was used to predict fungal ecological function traits, which primarily include three trophic modes of the fungal community: pathotroph (by harming host cells to receive nutrients), symbiotroph (by exchanging resources with host cells to receive nutrients), and saprotroph (by breaking down dead host cells to receive nutrients) (Tedersoo et al., 2014; Nguyen et al., 2016). The changing curves of AWCD values of the Biology ECO-plate were created by R software. The Tukey–Kramer of ANOVA was performed to test the differences in microbial functions, soil carbon, nitrogen fractions, soil enzyme activity, and soil organic acid contents among multiple groups. The bubble chart of soil factors was created by R packages of “ggpubr.” The heatmap between bacterial biogeochemical functions and family lever communities was generated by R packages of “reshape,” “psych,” “ggtree,” “dplyr,” “tidyverse,” and “aplot” (Yu et al., 2018; Liu et al., 2022; Revelle, 2022). The Mantel test was used to analyze the relationship among microbial communities at the phylum level and soil carbon and nitrogen fractions, soil enzyme activity, and soil organic acids contents with straw mulch.

The “piecewiseSEM” of the R package was used to create the structural equation model (SEM) among microbial structure and soil carbon and nitrogen fractions, soil enzyme activity, and soil organic acid contents (Lefcheck, 2016). The first principal component of principal coordinate analysis (PCoA) of microbial communities was used based on OTUs to represent the microbial community structure at the SEM (Yang et al., 2021). The SEM was created based on the following hypothesizes: (1) The bacterial and fungal structure changed with the increasing straw mulch amount; (2) the changed bacterial and fungal structure had different contributions to the soil carbon and nitrogen cycle; (3) the soil enzyme activity and organic acid content induced by the changing microbial community would affect soil carbon and nitrogen pools. Finally, we chose the goodness of fit model according to the *P*-value and Akaike information criterion (AIC) value of models.

## 3. Results

### 3.1. The structure of microorganism communities changed by increasing straw mulch amounts

**Figure 1:**
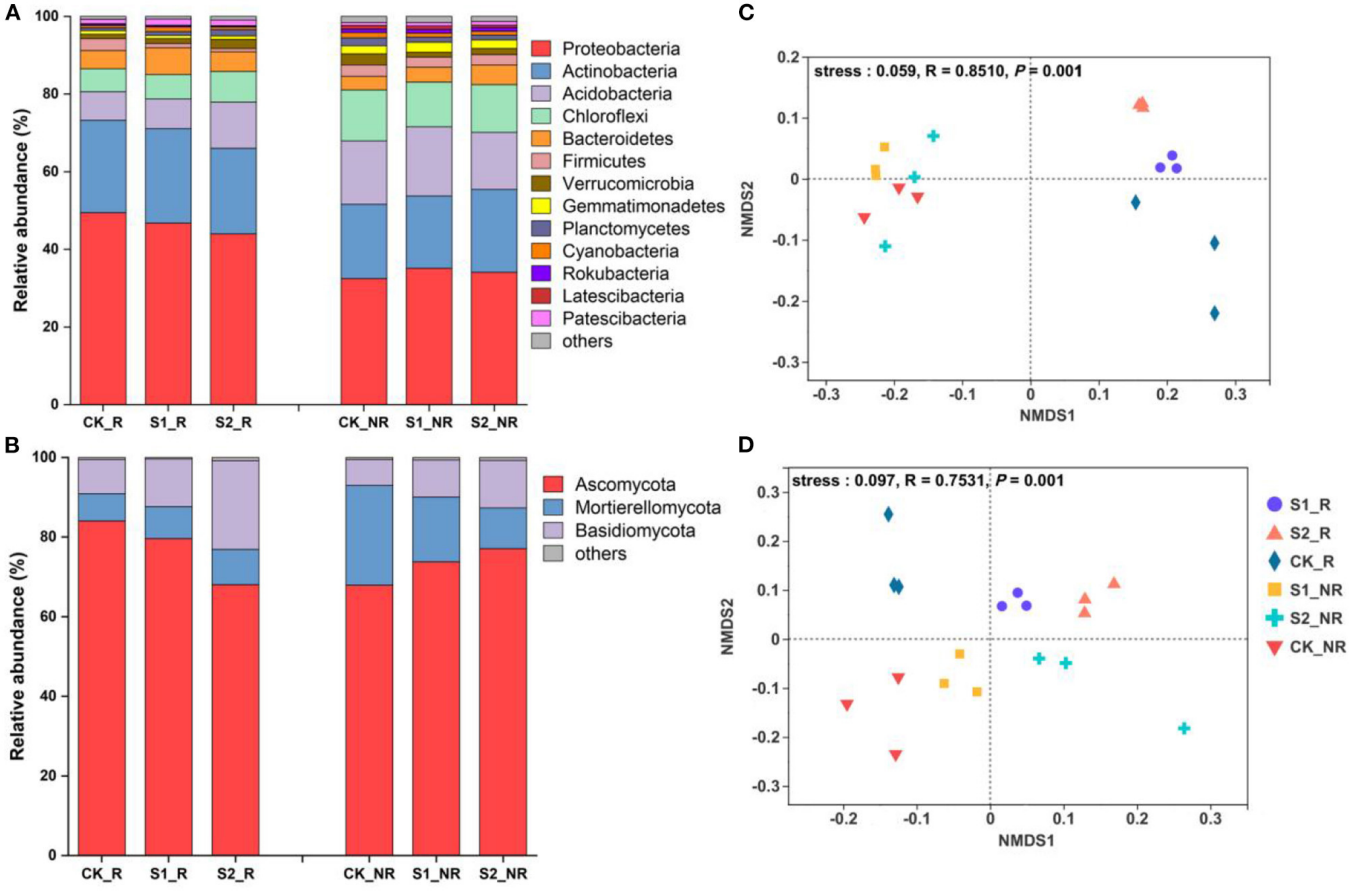
Spatial distribution characteristic of bacterial and fungal communities in the soil of the maize field. **(A)** The stacked bar of bacterial community relative abundance at the phylum level; **(B)** the stacked bar of fungal community relative abundance at the phylum level; **(C)** the non-metric multidimensional scaling (NMDS) analysis of bacteria at the OTU level; **(D)** the non-metric multidimensional scaling (NMDS) analysis of fungi at the OTU level. The “others” in **(A)** and **(B)** represent the summary of the relative abundance of less than 1% of species. S1_R, S2_R, and CK_R represent the rhizosphere soil of treatments S1, S2, and CK, respectively; S1_NR, S2_NR, and CK_NR represent the non-rhizosphere soil of treatments S1, S2, and CK, respectively (same below in all figures and tables).

Within-sample diversity data revealed that two different quantities of straw mulch enhanced alpha diversity of the microbial community in maize rhizosphere and non-rhizosphere soil although not significantly (*P* = 0.63 and *P* = 0.067, respectively; Table 1; Supplementary Table S1). Otherwise, there was a significant difference in microbial community richness in maize non-rhizosphere soil (*P* = 0.048; Table 1). Furthermore, the NMDS analysis results of microbiological community abundance illustrated that the bacterial and fungal communities were reshaped remarkably into different individual groups by straw mulch amounts in maize rhizosphere and non-rhizosphere (*P* = 0.01), which were separated along the first coordinate axis (Figures 1C, D), indicating that both straw mulch and its amount disturbed the microbiota in maize rhizosphere and non-rhizosphere. In addition, the Adonis analysis results demonstrated that bacterial and fungal community compositions changed significantly by the increasing straw mulch amount in the maize rhizosphere (*R*^2^ = 0.56, *P* = 0.005; *R*^2^ = 0.65, *P* = 0.002) and non-rhizosphere soil (*R*^2^ = 0.35, *P* = 0.004; *R*^2^ = 0.47, *P* = 0.009; Table 1). Moreover, the volcano plot results of enrichment and depletion OTUs revealed that increasing straw mulch amounts increased the upregulated and downregulated population numbers of bacterial (Supplementary Figures S1A–C) and fungal (Supplementary Figures S1D–F) communities in maize rhizosphere soil. The fungal population in the non-rhizosphere exhibited the same results (Supplementary Figures S1J–L). In addition, the influence of straw mulch amount on microbial population structure was stronger for fungi than bacteria in maize rhizosphere and non-rhizosphere soil (Supplementary Figure S1).

**Table 1:** Adonis analysis of the effect of the straw mulch on the microbial community composition and diversity in the maize rhizosphere and non-rhizosphere soil environment based on OTU abundance.

	**Bacterial community structure**		**Fungal community structure**		**Microbial community diversity**		**Microbial community richness**	
	*R* ^2^	***P*** **-value**	*R* ^2^	***P*** **-value**	*R* ^2^	***P*** **-value**	*R* ^2^	***P*** **-value**
Rhizosphere	0.562	0.005^**^	0.649	0.002^**^	0.212	0.63	0.119	0.884
Non-rhizosphere	0.345	0.004^**^	0.467	0.009^**^	0.491	0.067	0.545	0.048^*^

^*^0.01 < *P* ≤ 0.05.

^**^0.001 < *P* ≤ 0.01.

The difference in maize soil microbiota among the straw mulch treatments was significant and detectable at the phylum level. Raising straw mulch amounts reduced the relative abundances of *Proteobacteria* and *Firmicutes* while increasing the relative abundances of *Acidobacteria, Chloroflexi, Verrucomicrobia*, and *Planctomycetes* in maize rhizosphere soil (Figure 1A). More specifically, the relative abundance of *Bacteroidetes* in treatment S1 was considerably greater than CK (*P* = 0.04) and S2 (*P* = 0.005), whereas the relative abundance of *Firmicutes* in treatment S2 was significantly lower than S1 (*P* = 0.04) and CK (*P* = 0.003) in maize rhizosphere soil (Figure 1A; Supplementary Table S2). Simultaneously, compared to CK, treatment S1 increased the relative abundance of *Gemmatimonadetes* (*P* = 0.04), whereas treatment S2 decreased the relative abundance of *Planctomycetes* (*P* = 0.04) in maize non-rhizosphere soil (Figure 1A; Supplementary Table S2). For the fungal community, in comparison with CK, the treatments S1 and S2 significantly reduced the relative abundance of *Ascomycota* (*P* = 0.04 and *P* = 0.001, respectively) while significantly increasing the relative abundance of *Basidiomycota* (*P* = 0.002 and *P* = 0.004, respectively) in maize rhizosphere soil (Figure 1B; Supplementary Table S3). Nevertheless, the relative abundance of *Ascomycota* and *Basidiomycota* in maize non-rhizosphere soil was raised marginally by the raising straw mulch amount (*P* > 0.05; Figure 1B; Supplementary Table S3).

Furthermore, the heatmap results of species relative abundance at the family level showed that the treatments S1 and S2 enriched more types of bacterial communities than CK in maize rhizosphere and non-rhizosphere soil (Supplementary Figures S2A, B). In particular, the treatments S1 and S2 increased the relative abundance of *Acetobacteraceae, Acidothermaceae, Caulobacteraceae, Chitinophagaceae, Rhodanobacteraceae*, and others in maize rhizosphere and non-rhizosphere soil while decreasing the relative abundance of *Moraxellaceae* (Supplementary Figures S2A, B). The treatment S2 was the most beneficial for enriching multiple kinds of the fungal community in the maize rhizosphere and non-rhizosphere soil, and it greatly enhanced the relative abundance of *Chaetomiaceae, Chaetosphaeriaceae, Hydnodontaceae, Trichosphaeriaceae, Hypocreales_fam_Incertae_sedis*, and others (Supplementary Figures S2C, D).

### 3.2. The regulation of soil carbon and nitrogen components, enzyme activity, and organic acids by straw mulch

**Figure 2:**
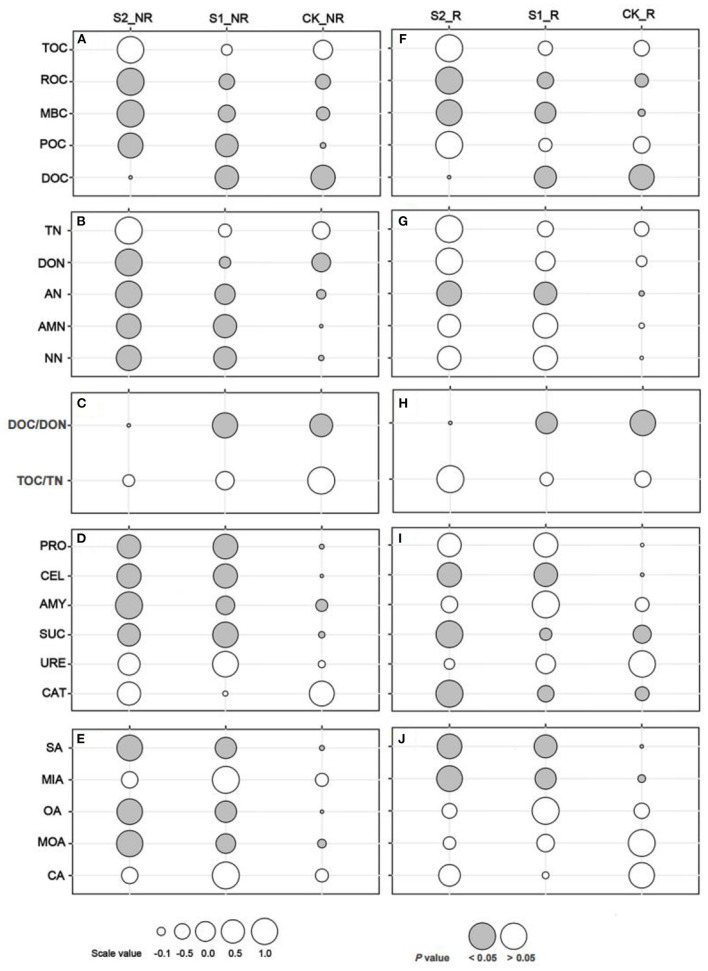
One-way ANOVA multiple comparisons (Tukey) of soil organic carbon and nitrogen components, enzyme activities, and organic acid contents. **(A–E)** shows the comparison of soil carbon fractions, nitrogen fractions, C/N ratio, enzyme activities, and organic acid contents in maize non-rhizosphere soil, respectively; (**F–J)** show the comparison of soil carbon fractions, nitrogen fractions, C/N ratio, enzyme activities, and organic acid contents in maize rhizosphere soil, respectively. The gray bubble represents significant differences at the *P* <0.05 level, and the size of the bubble represents the scale value. TOC, total organic carbon; MBC, microbial biomass carbon; POC, particulate organic carbon; ROC, readily oxidizable organic carbon; DOC, dissolved organic carbon; TN, total nitrogen; AMN, ammonium nitrogen; AN, available nitrogen; NN, nitrate nitrogen; DON, dissolved organic nitrogen; DOC/DON, the ratio of DOC to DON; TOC/TN, the ratio of TOC to TN; PRO, protease; CEL, cellulase; AMY, amylase; SUC, sucrase; URE, urease; CAT, catalase; OA, oxalic acid; MOA, malonic acid; MIA, malic acid; CA, citric acid; SA, succinic acid (the same below in all figures and tables).

Our study demonstrated that applying and increasing straw mulch amounts cannot significantly change soil TN and TOC in maize rhizosphere and non-rhizosphere soils, as well as their ratio (*P* > 0.05; Figures 2A–C, F–H). Otherwise, the treatment S2 significantly increased the labile C fractions of ROC, MBC, and POC (Figure 2A) and labile N fractions of DON, AN, AMN, and NN (Figure 2C) in maize non-rhizosphere soil (*P* < 0.05). Simultaneously, treatments S1 and S2 substantially enhanced those soil enzymes related to C or N degradation such as protease, cellulase, amylase, and sucrase activities in the maize non-rhizosphere soil compared to CK (*P* < 0.05; Figure 2D). Compared with maize non-rhizosphere soil, the straw mulch had a smaller effect on labile C and N fractions and related enzyme activities in maize rhizosphere soil. In detail, the ROC, MBC (Figure 2F), and AN (Figure 2G) in the maize rhizosphere were outstandingly raised by the increasing straw mulch amount (*P* < 0.05). On the contrary, the DOC (Figures 2A, F) and DOC/DON (Figures 2C, H) ratio markedly decreased by the increasing straw mulch amounts in maize rhizosphere and non-rhizosphere soil (*P* < 0.05). In addition, in maize rhizosphere soil, the cellulase, sucrase, and catalase activities of the treatment S2 were significantly higher than CK (*P* < 0.05), whereas straw mulch had no significant effect on soil amylase, protease, or urease activity (Figure 2I). Furthermore, the content of succinic acid, oxalic acid, and malonic acid outstanding improved with increasing straw mulch amounts in maize non-rhizosphere (*P* < 0.05; Figure 2E), as well as the content of malic acid and succinic acid in maize rhizosphere (*P* < 0.05; Figure 2J).

### 3.3. The functional characteristics of the microbiological community with straw mulch in maize soil

**Figure 3:**
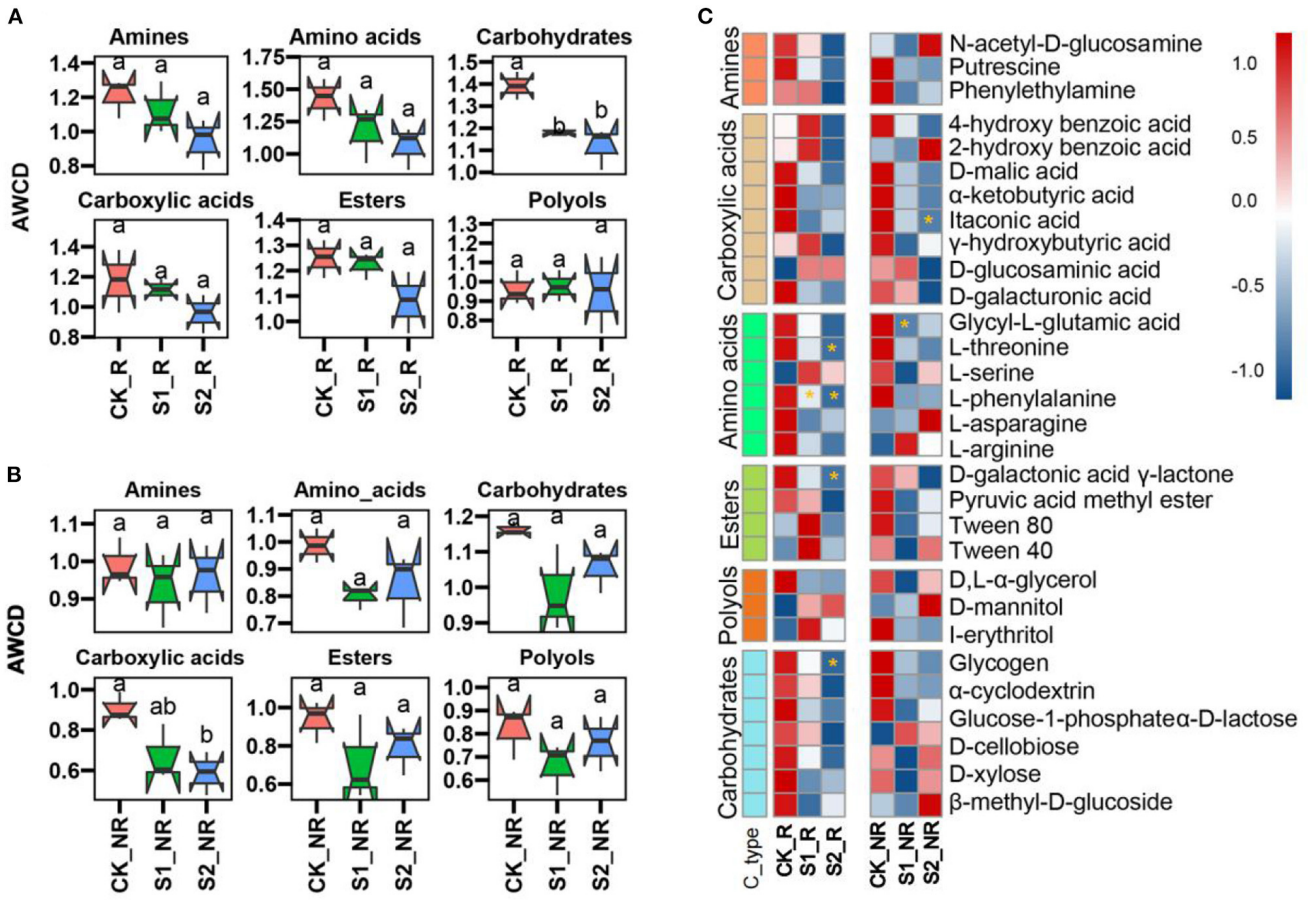
Carbon resource metabolism functions of the microbial community under straw mulch. **(A, B)** The carbon-containing compound metabolism ratio of the microbial community with straw mulch in maize rhizosphere and non-rhizosphere soil, respectively. The different lower cases mean significant differences among treatments. **(C)** The 31 kinds of carbon resource metabolism efficiency of a microbial community with straw mulch in the rhizosphere and non-rhizosphere soil (“*” represents a significant difference compared with CK at *P* < 0.05 level).

We assessed the carbon source consumption efficiency of the microbial community, which was boosted by varying the quantity of straw mulch used (Figure 3; Supplementary Figure S3). The results showed that increasing the amount of straw mulch lowered the microbial utilization ratio of carbohydrates, esters, amino acids, carboxylic acids, and amines in maize rhizosphere and non-rhizosphere soil (Figures 3A, B). Compared with CK, the treatments S1 and S2 significantly decreased the carbohydrate metabolism ratio of microorganisms in maize rhizosphere soil (*P* < 0.05; Figure 3A), and the treatment S2 remarkably cut down the carboxylic acid metabolism ratio of microorganisms in maize non-rhizosphere soil (*P* < 0.05; Figure 3B). More specifically, compared with CK, the S1 treatment remarkably reduced the metabolism ratio of L-phenylalanine and glycyl-L-glutamic acid by microorganisms in maize rhizosphere and non-rhizosphere, respectively (*P* < 0.05; Figure 3C). The treatment S2 significantly decreased the utilization ratio of glycogen, D-galactonic acid γ-lactone, L-phenylalanine, and L-threonine by microorganisms in maize rhizosphere and α-ketobutyric acid in maize non-rhizosphere soil (*P* < 0.05; Figure 3C).

**Figure 4:**
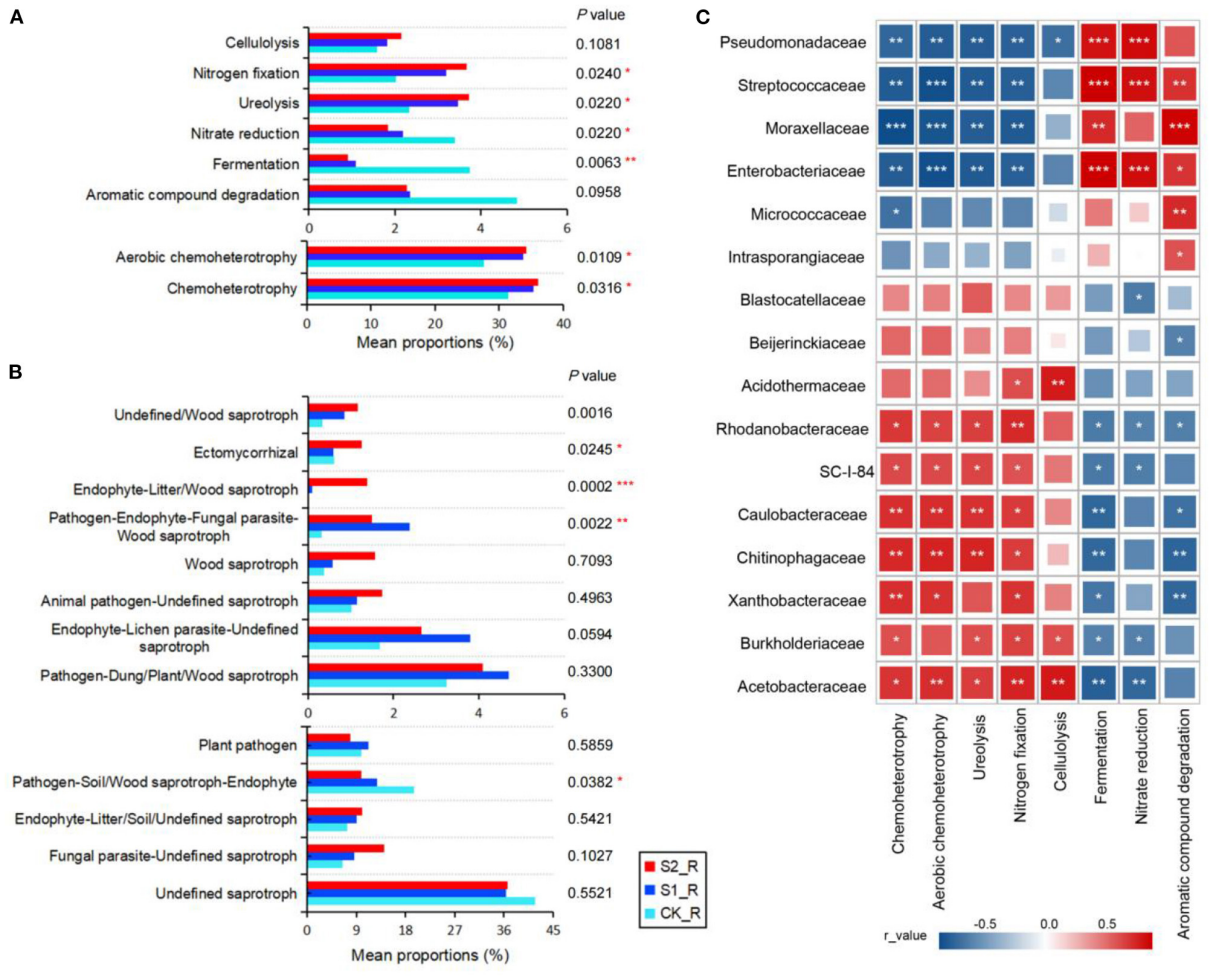
Functional predictions of the microbial community with straw mulch in maize rhizosphere soil. **(A)** Bacterial function annotation by FAPROTAX with straw mulch in maize rhizosphere soil (the mean proportions >1% were shown); **(B)** function annotation of a fungal guild type based on FUNGuild with straw mulch in maize rhizosphere soil (the mean proportions of >1% were shown); **(C)** the correlation heatmap graph between bacterial environment functions and species (family level, the relative abundance >1% was shown) in maize rhizosphere soil with straw mulch. “*,” “**,” and “***” represent significant differences at *P* < 0.05, 0.01 < *P* < 0.05, and 0.001 < *P*< 0.01 level, respectively. The Benjamini–Hochberg method was used to correct *P*-values.

This study analyzed the functions of the bacterial biogeochemical matter cycle based on the FAPROTAX database. The results showed that performing straw mulch remarkably promoted the processes of chemoheterotrophy, aerobic chemoheterotrophy, ureolysis, and nitrogen fixation of the bacterial community in maize rhizosphere soil, while significantly inhibiting the processes of fermentation and nitrate reduction (*P* < 0.05; Figure 4A; Supplementary Table S4). The cellulolysis was also marginally raised by increasing the straw mulch amount in maize rhizosphere and non-rhizosphere soil (Figure 4A; Supplementary Figure S5A). Nonetheless, there was no substantial variation in these bacterial community functions in maize non-rhizosphere soil with straw mulch application (*P* > 0.05; Supplementary Figure S5A; Supplementary Table S5). Furthermore, the Pearson correlation analysis was performed between dominant bacterial communities and the environmental functions (Figure 4C; Supplementary Figure S5C). The findings revealed that chemoheterotrophy, aerobic chemoheterotrophy, ureolysis, nitrogen fixation, and cellulolysis had a significant positive correlation with the relative abundance of *Acetobacteraceae, Burkholderiaceae, Xanthobacteraceae, Chitinophagaceae, Caulobacteraceae, SC-I-84*, and *Rhodanobacteraceae* while having a significant negative correlation with the relative abundance of *Enterobacteriaceae, Moraxellaceae, Streptococcaceae*, and *Pseudomonadaceae* in the maize rhizosphere (*P* < 0.05; Figure 4C). Nevertheless, the fermentation, nitrate reduction, and aromatic compound degradation all showed contrary associations with the aforementioned species compared with the above functions (*P* < 0.05; Figure 4C). However, the correlation between environmental functions and multiple bacterial populations in maize non-rhizosphere did not alter considerably (Supplementary Figure S5C).

Simultaneously, the fungal trophic functions were analyzed by the FUNGuild database. We observed that the main trophic type of the fungal community was saprotroph in maize rhizosphere and non-rhizosphere soil (Supplementary Figure S4). After straw mulch, the endophyte and litter or wood saprotroph proportions raised outstanding in maize rhizosphere and non-rhizosphere soil (*P* < 0.01; Figure 4B; Supplementary Figure S5B; Supplementary Tables S6, S7). In addition, according to the significant differences in fungal guild type, the corresponding fungal communities in maize rhizosphere soil were selected (Supplementary Figure S6). The results exhibited that straw mulch application increased the relative abundance of *Chaetomiaceae, Chaetosphaeriaceae*, and *Hypocreales_fam_Incertae_sedis*, leading to the fungus of litter or wood saprotroph growing in maize rhizosphere soil (Supplementary Figure S6).

### 3.4. The relationship between soil factors and microbial communities regulated by straw mulch

**Figure 5:**
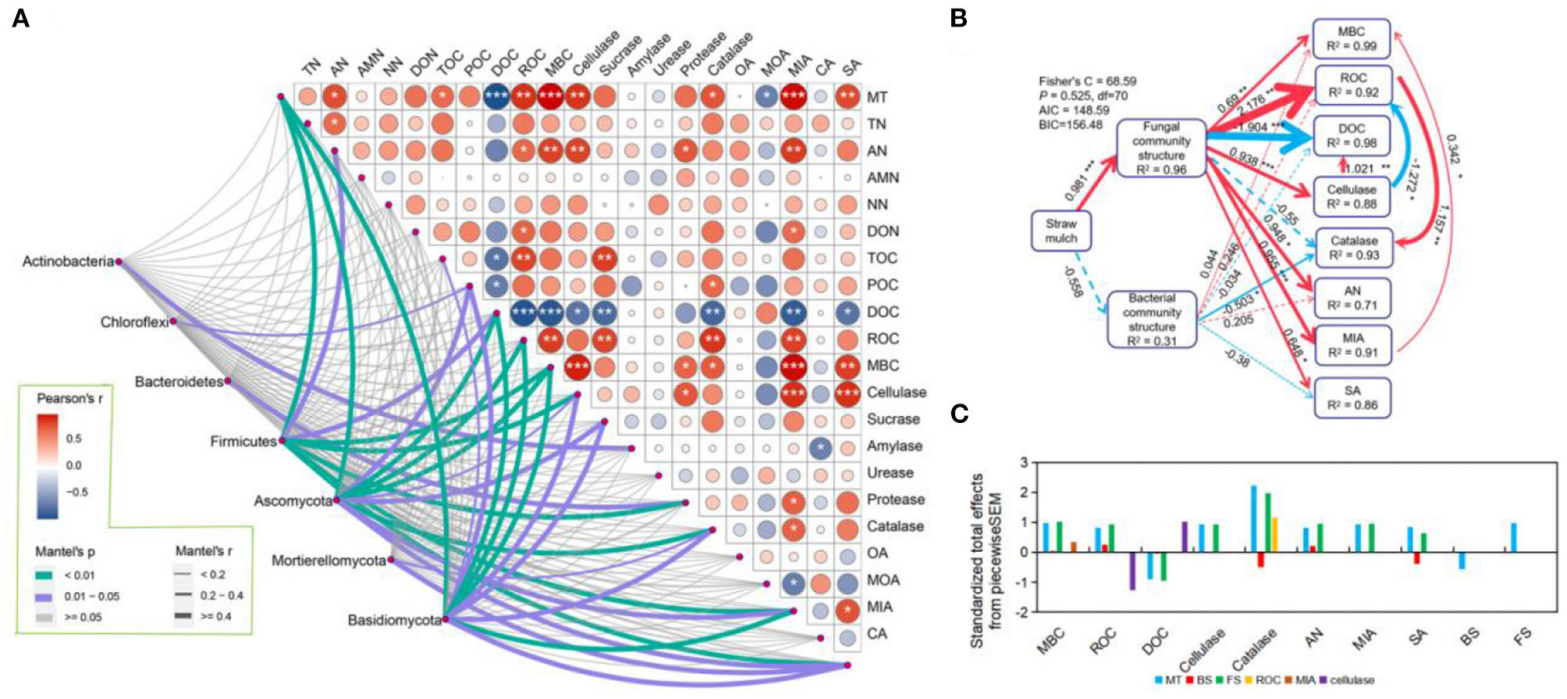
Relationship between the microbial community and soil factors in maize rhizosphere soil with straw mulch. **(A)** The correlation between microbial community (phylum level) and soil factors in maize rhizosphere soil with straw mulch. The right upper shows the Pearson correlation among components of soil carbon and nitrogen, enzyme activity, and organic acids. The left bottom shows the Mantel analysis between the microbial community and soil factors. The width and color of the lines show different Mantel's *r* and *P*-values. Just present the microbial community that has a significant correlation. **(B)** The piecewise structural equation models (SEMs) show the direct and indirect correlation among straw mulch, microbial community structure (Bray–Curtis distance), and soil factors in maize rhizosphere soil with straw mulch. Solid and dashed lines with arrows denote significant and non-significant correlations, respectively. Red and blue lines with an arrow indicate positive and negative effects, respectively. The width of lines with an arrow shows the standardized path coefficients, and the special values are labeled on the adjacent lines. The value of R^2^ in boxes denotes the response explained variance. **(C)** The standardized total effects among straw mulch, bacterial community structure, and soil factors are calculated with piecewise structure equation models. MT, straw mulch treatments; TN, total nitrogen; AN, available nitrogen; AMN, ammonium nitrogen; NN, nitrate nitrogen; DOC, dissolved organic nitrogen; TOC, total organic nitrogen; POC, particulate organic nitrogen; ROC, readily oxidizable organic carbon; MBC, microbial biomass carbon; OA, oxalic acid; MOA, malonic acid; MIA, malic acid; CA, citric acid; SA, succinic acid. Significant difference levels: ***P < 0.001, **P < 0.01, and *P < 0.05.

The results of the Pearson test showed that straw mulch (MT) had a significant positive correlation with the contents of AN, TOC, ROC, MBC, MIA, SA, and activity of cellulase and catalase in maize rhizosphere soil but a negative correlation with DOC content (*P* < 0.05; Figure 5A; Supplementary Table S8). The Mantel test results denoted that these soil factors exhibited a significant correlation with the abundance of microbial communities regulated by straw mulch, particularly *Firmicutes, Ascomycota*, and *Basidiomycota* (*P* < 0.05; Figure 5A; Supplementary Table S10). Similar results were presented in maize non-rhizosphere (*P* < 0.05; Supplementary Figure S7A; Supplementary Tables S9, S10). Hence, we created the piecewise structure equation model based on the Pearson and Mantel test among straw mulch, soil factors, and the microbial community. The model revealed that the straw mulch dominated the fungal community structures to drive the cycle of soil labile C and N (Figure 5B; Supplementary Figure S7B). Straw mulch, in particular, favorably controlled the fungal community structures (*R*^2^ = 0.96, *P* < 0.001), considerably increasing the levels of MBC, ROC, AN, MIA, SA, and cellulase activity in the maize rhizosphere soil (*P* < 0.05; Figure 5B). In addition, the fungal community structures changed by straw mulch positively regulated MBC and DOC indirectly by improving MIA content and cellulase activity in the maize rhizosphere soil, respectively. Otherwise, in the maize rhizosphere soil, the fungal community structure had a direct negative and positive impact on the soil's DOC and ROC contents, respectively, and it directly enhanced soil cellulase activity to indirectly lower ROC contents (Figure 5B). In general, the total effect of the straw mulch on catalase activity was the highest (Figure 5C). The straw mulch positively regulated fungal community structure directly and then indirectly positively drove ROC content to improve catalase activity (Figure 5B).

For the maize non-rhizosphere soil, the regulation of microbial community structures changed by straw mulch was weaker than in rhizosphere soil, and it was the same for soil C and N components (Figure 5; Supplementary Figure S7). The straw mulch had a positive correlation with fungal community structure in non-rhizosphere soil (*R*^2^ = 0.92, *P* < 0.001; Supplementary Figure S7), whereas the fungal community structure had a significant direct positive effect on the activity of amylase and protease, along with AN and NN content, and indirectly affected AMN by regulating protease (*P* < 0.05; Supplementary Figure S7). Meanwhile, the fungal community structure indirectly improved MBC content and cellulase activity by enhancing amylase and protease activity in the non-rhizosphere soil, respectively (*P* < 0.05; Supplementary Figure S7). The total effect of piecewiseSEM revealed that straw mulch had a detrimental influence on DOC content in the non-rhizosphere. This result indicated that straw mulch principal positively regulated fungal community structure, thereby improving the amylase activity to enhance the MBC fraction and reduce DOC content (Supplementary Figures S7B, C).

## 4. Discussion

### 4.1. Straw mulch changes the soil microbial community structure in the maize field

In our study, straw mulch marginally increased the alpha diversity of bacterial and fungal communities in maize rhizosphere and non-rhizosphere soil but not significantly. It is consistent with previous studies that found straw mulch had no effect on alpha diversity indices of soil bacterial and fungal communities but changed the microbial community composition (Zheng et al., 2018; Liu et al., 2021). This suggests that applying straw mulch for the short term will not disrupt the balance of soil native microbial species diversity. Otherwise, increasing the quantity of straw mulch dramatically affected the bacterial and fungal community structures and produced various independent microbial groups in the maize rhizosphere and non-rhizosphere soil. Similarly, recent research indicated that straw mulch significantly altered the organization of the soil microbial population in the wheat and rape field (Zhou et al., 2019; Liu et al., 2022). We further observed that the upregulated and downregulated population numbers of the microbial community were raised by increasing straw mulch amounts in both the maize rhizosphere and non-rhizosphere, in line with previous research that the change in microbial community structure induced by straw mulch amounts may be a foodweb competition mechanism because straw provides multiple nutrition matters such as the input of carbon and nitrogen (Kou et al., 2020; Song et al., 2020). Generally, fungi primarily decompose recalcitrant soil organic matter, while bacteria are more vital in the mineralization of labile fractions (Lindahl and Tunlid, 2015; Fu et al., 2019). As a result, fungi showed significant changes in species abundance under straw mulch than bacteria because fungal communities are adept at degrading lignin, cellulose, and hemicellulose, all of which are abundant in wheat straw (Cong et al., 2020; Tufail et al., 2021).

At the bacterial phylum level, we observed that the relative abundance of *Firmicutes* was significantly reduced from 3 to 0.87% by increasing the straw mulch amount in the soil of the maize field, which could produce urease and/or catalase and promote soil nitrogen cycle (Ren et al., 2018; Zhang et al., 2021). At the fungal phylum level, most previous studies confirmed that the *Ascomycota*, along with a few basidiomycetous fungi, dominate during the early stages of litter decay, with the *Ascomycota* gradually being replaced by the *Basidiomycota* during the later stages of litter decomposition (Frankland, 1998; Osono and Takeda, 2002; Osono, 2007; Eichlerová et al., 2015). During the litter degradation process, synergistic matters that relay between *Ascomycota* and *Basidiomycota* may be triggered by breakdown products induced at different stages (Taylor, 2001; Watkinson, 2016). Similarly, we found that the relative abundance of *Ascomycota* significantly decreased from 81 to 66% by increasing the straw mulch amount in the soil of the maize field, whereas the relative abundance of *Basidiomycota* significantly increased from 8 to 22%, indicating that increasing straw mulch amount could accelerate the succession process of the fungal community in the soil of the maize field. In addition, earlier research has revealed that soil ecological niche is also a crucial factor for microbial community structure (Dumbrell et al., 2010). In line with prior research, we discovered that the bacterial and fungal community composition with straw mulch, as well as their succession rules, differed between maize rhizosphere and non-rhizosphere soil. It might be disturbed by the activity of the maize root system, such as the production of root-system excretions, resulting in a soil ecological environment distinction between the rhizosphere and non-rhizosphere (Khan et al., 2009).

### 4.2. Straw mulch promotes the nitrogen and carbon cycling functions of the soil microbial community in the maize field

Wheat straws are rich in C and N fractions, including cellulose, hemicellulose, lignin, and protein (Khan and Mubeen, 2012; Zhong et al., 2020). Therefore, applying wheat straw mulch can reshape the soil microbial food web due to the input of C and N sources, sequentially leading to a change in the community composition and function of the microbiome (Mendes et al., 2014; Cobellis et al., 2016). In our study, straw mulch significantly increased the processes of bacterial chemoheterotrophy and aerobic chemoheterotrophy in maize rhizosphere soil, proving that a large number of bacteria obtain carbon and energy by oxidizing organic compounds of straw for their growth (Yu et al., 2021), and implying that straw mulch can stimulate the soil carbon cycle by providing food sources for the bacterial community. Additionally, our study demonstrated that straw mulch outstanding reduced aromatic compound degradation and fermentation bacteria in maize rhizosphere soil, indicating that the bacterial community modified by straw mulch can reduce carbon dioxide and methane emissions, thus benefiting soil carbon fix (Miller and Wolin, 1981; Wang et al., 2020). In addition, soil bacteria affect the soil nitrogen cycle by mediating nitrogen fixation, ammonification, denitrification, nitrification, and nitrate reduction (Yoon et al., 2015). Our study observed that straw mulch remarkably enhanced ureolysis and nitrogen fixation of bacteria in maize rhizosphere soil, whereas it significantly inhibited nitrate reduction. This suggests that straw mulch drives the soil nitrogen cycle by bacteria via two mechanisms. For one thing, straw mulch promoted bacterial ureolysis and nitrogen fixation to increase ammonia by degradation of organic nitrogen compounds such as urea and fixing dinitrogen, respectively (Burton and Prosser, 2001; Kuypers et al., 2018). Then, ammonia can fix ammonium nitrogen and nitrate nitrogen by hydrolysis and oxidization, respectively (Jiang et al., 2016; Kuypers et al., 2018). For another, straw mulch reduced the nitrate reduction of bacteria, thus improving nitrate-nitrogen accumulation in soil (Kuypers et al., 2018). It may be the reason that the content of ammonium and nitrate nitrogen in S1 and S2 was significantly higher than CK in maize non-rhizosphere soil (Figure 2B). Furthermore, the ammonium and nitrate nitrogen that accumulate in the maize rhizosphere soil might be absorbed by maize to promote the nitrogen cycle between soil and plant (Jackson et al., 1989; Li et al., 2013); hence, their contents differed insignificantly across treatments. In addition, most previous studies have illustrated that *Proteobacteria, Firmicutes*, and *Bacteroidetes* of bacteria are mostly responsible for driving soil carbon and nitrogen cycle (He et al., 2012; Ren et al., 2018; Zhang et al., 2021). Consistent with previous findings, we discovered that straw mulch accelerated N and C cycling in maize rhizosphere soil by regulating the bacterial community ratio, primarily including *Burkholderiaceae* (γ*-Proteobacteria*), *Xanthobacteraceae* (α*-Proteobacteria*), *Chitinophagaceae* (*Bacteroidia*), *Rhodanobacteraceae* (γ*-Proteobacteria*), *Caulobacteraceae* (α*-Proteobacteria*), *Acetobacteraceae* (α*-Proteobacteria*), *Enterobacteriaceae* (γ*-Proteobacteria*), and *Streptococcaceae* (*Firmicutes*). These bacteria can enhance the processes of chemoheterotrophy, aerobic chemoheterotrophy, ureolysis, cellulolysis, and nitrogen fixation and inhibit the processes of aromatic compound degradation, fermentation, and nitrate reduction, thereby promoting C and N cycle.

In the meantime, the results of the fungal community functional predictor revealed that adding straw mulch significantly increased the proportion of wood saprotrophs in maize rhizosphere soil, such as *Chaetomiaceae* and *Chaetosphaeriaceae*, which could regulate the C cycle by degrading cellulose, hemicellulose, and lignin (Kuramae et al., 2013; Zhang C. et al., 2020; Ferrari et al., 2021). This finding agrees with a recent study that found straw cellulose, hemicellulose, and lignin to be the key food web components that dominated the fungal community structure (He et al., 2022).

Additionally, the results of adding carbon sources incubation showed that the carbon metabolism ratio of the microbial community decreased by the increasing straw mulch amount, especially carbohydrates. There could be two reasons for this outcome. For one thing, straw mulch impacted the fungal community composition more than bacteria in our study, which benefits the metabolism of recalcitrant carbon sources rather than that of soluble carbon sources (Zhong et al., 2018; Cong et al., 2020). For another, the decomposition of straw mulch supplied adequate carbon for the microbial community to flourish, thus reducing their capacity to predation the carbon resource, which implied that more carbon components would be maintained in the soil environment (Huo et al., 2017; Hu et al., 2021). Furthermore, Zhang X. et al. (2020) demonstrated that root hair proliferation strongly influenced enzyme activities, exudate release, and microbial substrate utilization. Consistent with this study, we found that the carbon source metabolism ratio of the microbial community in the rhizosphere soil was higher than in the non-rhizosphere soil, illustrating that the maize root system with straw mulch may promote soil carbon consumption by driving microbial community activity.

### 4.3. The process of interaction among soil carbon, nitrogen, and microbial populations with straw mulch

Straw mulch delivers diverse nutrient matters to the soil via microorganism degradation and mineralization, and a series of biogeochemical events would vary the ratio of distinct soil C and N components, boosting the soil matter cycle (Guo and Gifford, 2002; Leifeld and Kögel-Knabner, 2005; Yang et al., 2018; Jin et al., 2020). Our study demonstrated that straw mulch mediated the soil C and N fractions by regulating microbial community structures such as *Firmicutes, Ascomycota*, and *Basidiomycota*. These microbial communities were reported to be dramatically correlated with SOC fractions (Cong et al., 2020). Previous studies have shown that increasing carbon sequestration could improve soil quality and health while reducing greenhouse gas emissions (Chen et al., 2009; Wang et al., 2018). In our study, soil TOC content was marginally increased by straw mulch (*P* > 0.05), indicating that the short-term straw mulch had no significant contribution to changing the soil's stable C pool. Whereas straw mulch significantly increased MBC and ROC content while reducing DOC content in maize rhizosphere and non-rhizosphere soil (*P* < 0.05), these C fractions have been identified as labile soil organic C pools and critical markers of soil quality (Chen et al., 2009; Strosser, 2010). Despite this, the networks of straw mulch that governed labile C pools differed across maize rhizosphere and non-rhizosphere soil. In maize rhizosphere soil, straw mulch positively promoted fungal community structure, thus indirectly positively mediating MBC and ROC while negatively influencing DOC. Otherwise, straw mulch indirectly positively regulated MBC by the positively affected fungal and bacterial community structure in maize non-rhizosphere soil and then the MBC negatively affected DOC. These distinction networks may be related to frequent reports that show plants interact with soil to shape the rhizosphere microbial community, thus changing community functions (Minz et al., 2013; Zolla et al., 2013; Bakker et al., 2015). Additionally, recent research has shown that the fungus *Ascomycota* and *Basidiomycota* degrade cellulose by producing multiple cellulases (Kumar et al., 2008; Berka et al., 2011; Strakowska et al., 2014). Similarly, our findings reveal that the dominating fungal communities, *Ascomycota* and *Basidiomycota*, altered considerably with straw mulch quantity in maize rhizosphere soil, thereby indirectly regulating ROC and DOC content by directly improving cellulase activity. Furthermore, Chowdhury et al. (2014) indicated that malic acid content was positively associated with soil labile C levels. Consistent with the previous study, we illustrated that straw mulch remarkably enhanced malic acid content by fungal communities intervening in maize rhizosphere soil, thus having a large indirect influence on MBC content. Otherwise, straw mulch had an indirect positive effect on MBC content in maize non-rhizosphere soil by regulating fungal community structure to stimulate amylase activity, which could directly participate in the metabolic process of soil organic matter by catalyzing starch hydrolysis (Sherene, 2017; Ali et al., 2019).

Ammonium and nitrate N are substantial and effective N donors which can be directly taken in by plants (Loo, 1931; Caicedo et al., 2000; Wang et al., 2015). We discovered that the amount of ammonium and nitrate N rose significantly with straw mulch in maize non-rhizosphere soil, but there was no significant difference in rhizosphere soil, although both their contents were greater in rhizosphere soil than in non-rhizosphere soil. This finding suggested that straw mulch could provide maize with extra utilizable nitrogen by enhancing ammonium and nitrate N generation and that the maize root system may promote the N cycle through the consumption of ammonium and nitrate N during the straw degradation process. Moreover, it is becoming increasingly clear that *Ascomycota* and *Basidiomycota* populations contribute significantly to the biogeochemical nitrogen cycle in terrestrial ecosystems by participating in the mobilization and mineralization processes of ammonium and nitrate N (Montanini et al., 2002; Gorfer et al., 2011; Maeda et al., 2015). Similarly, we observed that straw mulch directly dominated the fungal community structure in maize non-rhizosphere soil, and as a result, the fungal community structure directly influenced nitrate N and indirectly affected ammonium N by directly impacting protease activity. According to Akhtar et al. (2018), favorable temperature and soil water conditions caused by straw mulch boosted soil available N, which was responsible for improved crop development. In our study, straw mulch indirectly drove the available nitrogen increase in maize rhizosphere and non-rhizosphere soil by directly promoting fungal community structure, especially *Ascomycota* and *Basidiomycota*, as well as the *Firmicutes* of bacteria, which carry genes coding for nitrogen cycles of urease (Zhang et al., 2021) and significantly correlated with the available nitrogen content in maize rhizosphere soil (*P* = 0.023). Otherwise, total nitrogen had no significant difference among treatments in maize rhizosphere and non-rhizosphere soil. Overall, straw mulch promoted labile C and N pools to drive the C and N cycle by reshaping the structure of *Firmicutes, Ascomycota*, and *Basidiomycota* communities in the soil of the maize field.

## 5. Conclusion

In this study, we found that straw mulch had no significant contribution to the diversity of the bacterial and fungal communities in maize rhizosphere and non-rhizosphere soil, but it substantially modified their community structure, with the fungal community changing more than the bacterial. In general, straw mulch primarily regulated the community structures of *Proteobacteria, Firmicutes*, and *Bacteroidetes* to accelerate the processes of chemoheterotrophy, aerobic chemoheterotrophy, ureolysis, cellulolysis, and nitrogen fixation while inhibiting the processes of aromatic compound degradation, fermentation, and nitrate reduction, thereby promoting C and N cycle in maize rhizosphere soil. At the same time, straw mulch enhanced the proportion of *Chaetomiaceae* and *Chaetosphaeriaceae* to increase fungal saprotroph, which benefited the decomposition of cellulose, hemicellulose, and lignin and regulated the C cycle in the soil. The bacterial and fungal communities, modified with straw mulch, generally had a lower capacity for carbon source predation. Furthermore, straw mulch controlled the relative abundance of *Firmicutes, Ascomycota*, and *Basidiomycota* communities, influencing soil labile N and C pools such as AN, AMN, NN, MBC, ROC, and DOC fractions. The modified microbial community structure can alter soil labile N and C fractions directly and indirectly by affecting the concentration of malic acid and the activity of cellulase, amylase, and protease, with the fungal community structure being the most important. In total, straw mulch primarily reshaped microbial community structures to promote the production and flux of labile C and N fractions, thereby driving C and N cycle in the soil of the maize field.

## Data availability statement

The data presented in the study are deposited in the NCBI repository (https://www.ncbi.nlm.nih.gov/), accession numbers are PRJNA973987 (SAMN35155348 - SAMN35155365) and PRJNA974221 (SAMN35176313 - SAMN35176330) for bacterial and fungal raw sequencing data, respectively.

## Author contributions

LW was primarily responsible for project application, experimental design, and paper revision. BL was heavily involved in the experiment's implementation and writing. YD was primarily responsible for the experiment's execution and proofreading. XC, XH, QB, YW, YZ, BZ, KZ, XT, MD, and XX were primarily involved in some of the experiments in this study. All authors contributed to the article and approved the submitted version.
