# Supplementary material for: Straw mulch improves soil carbon and nitrogen cycle by mediating microbial community structure and function in the maize field
Source: Front Microbiol. 2023 Jul 18;14:1217966. doi: 10.3389/fmicb.2023.1217966 (PMC10391546; doi:10.3389/fmicb.2023.1217966)
Supplement: Supplementary file 1 [file Data_Sheet_1.PDF]

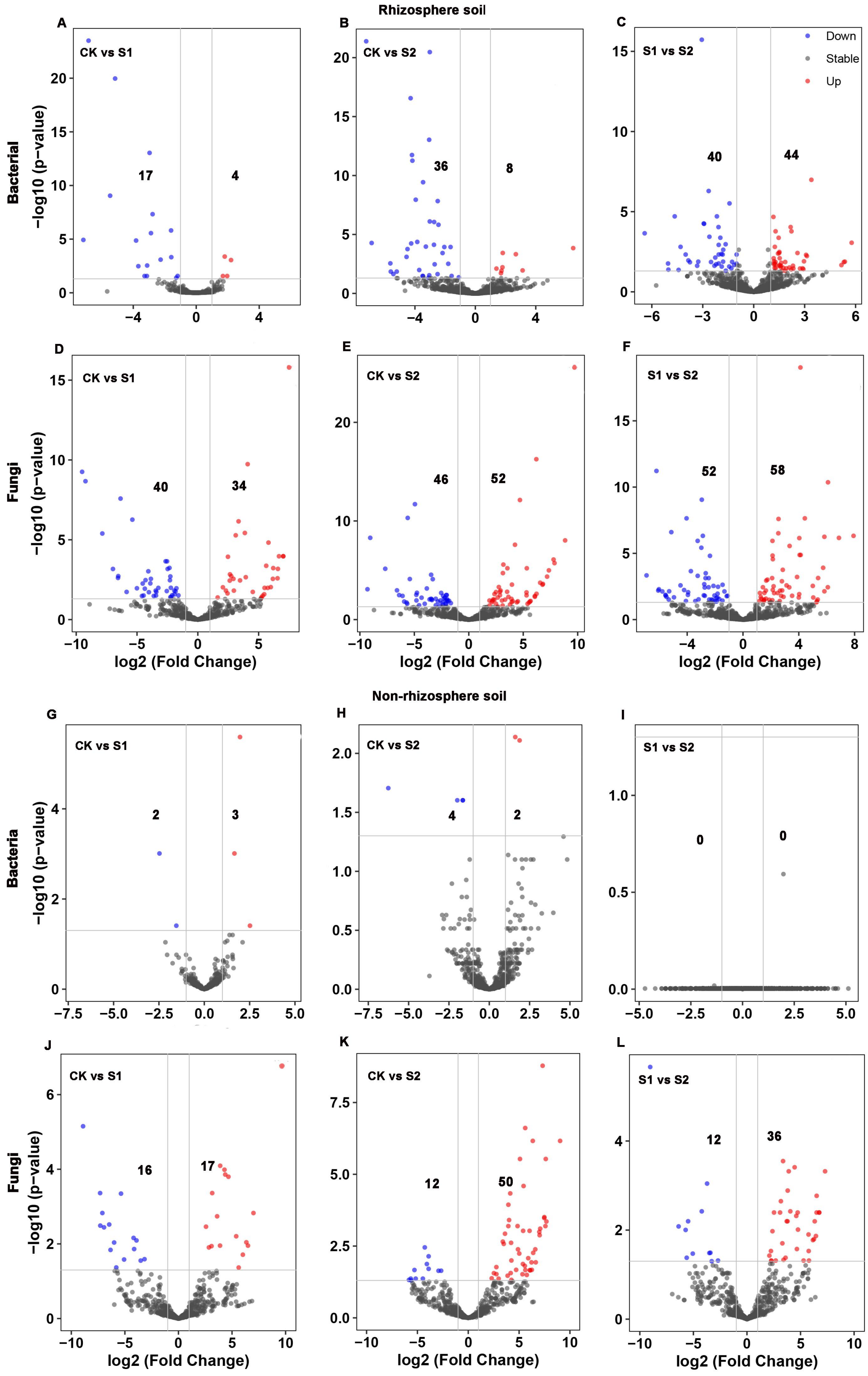

Rhizosphere soil
A
CK vs S1
17
4
B
CK vs S2
36
8
C
S1 vs S2
40
44
Down
Stable
Up
Bacterial
−log10 (p−value)
D
CK vs S1
40
34
E
CK vs S2
46
52
F
S1 vs S2
52
58
Fungi
−log10 (p−value)
log2 (Fold Change)
Non-rhizosphere soil
G
CK vs S1
2
3
H
CK vs S2
4
2
I
S1 vs S2
0
0
Bacteria
−log10 (p−value)
J
CK vs S1
16
17
K
CK vs S2
12
50
L
S1 vs S2
12
36
Fungi
−log10 (p−value)
log2 (Fold Change)

Fig. S1 The volcano plots show the enrichment and depletion of OTUs between different straw mulch amounts. The X-axis indicates the log2 fold of OTU abundance specific value between different straw mulch amounts, and the y-axis represents the -log10 scale of the FDR-corrected P-values. Each dot represents an individual OTU. The red and blue dots respectively represent OTU abundance up-regulated (adjust P-value < 0.05, log2 Fold Change >= 1) and down-regulated (adjust P-value < 0.05, log2 Fold Change <= -1) differences between the different straw mulch amounts. The gray dots represent the OTU abundance that has no significant difference between the straw mulch amounts. **A-C**): The bacterial flora abundance regulation of S1 compared with CK, S2 compared with CK, and S1 compared with S2 in maize non-rhizosphere soil, respectively; **D-F**) The fungal flora abundance regulation of S1 compared with CK, S2 compared with CK, and S1 compared with S2 in maize non-rhizosphere soil, respectively; **G-I**); The bacterial flora abundance regulation of S1 compared with CK, S2 compared with CK, and S1 compared with S2 in maize non-rhizosphere soil, respectively; **J-L**) The fungal flora abundance regulation of S1 compared with CK, S2 compared with CK, and S1 compared with S2 in maize non-rhizosphere soil, respectively.

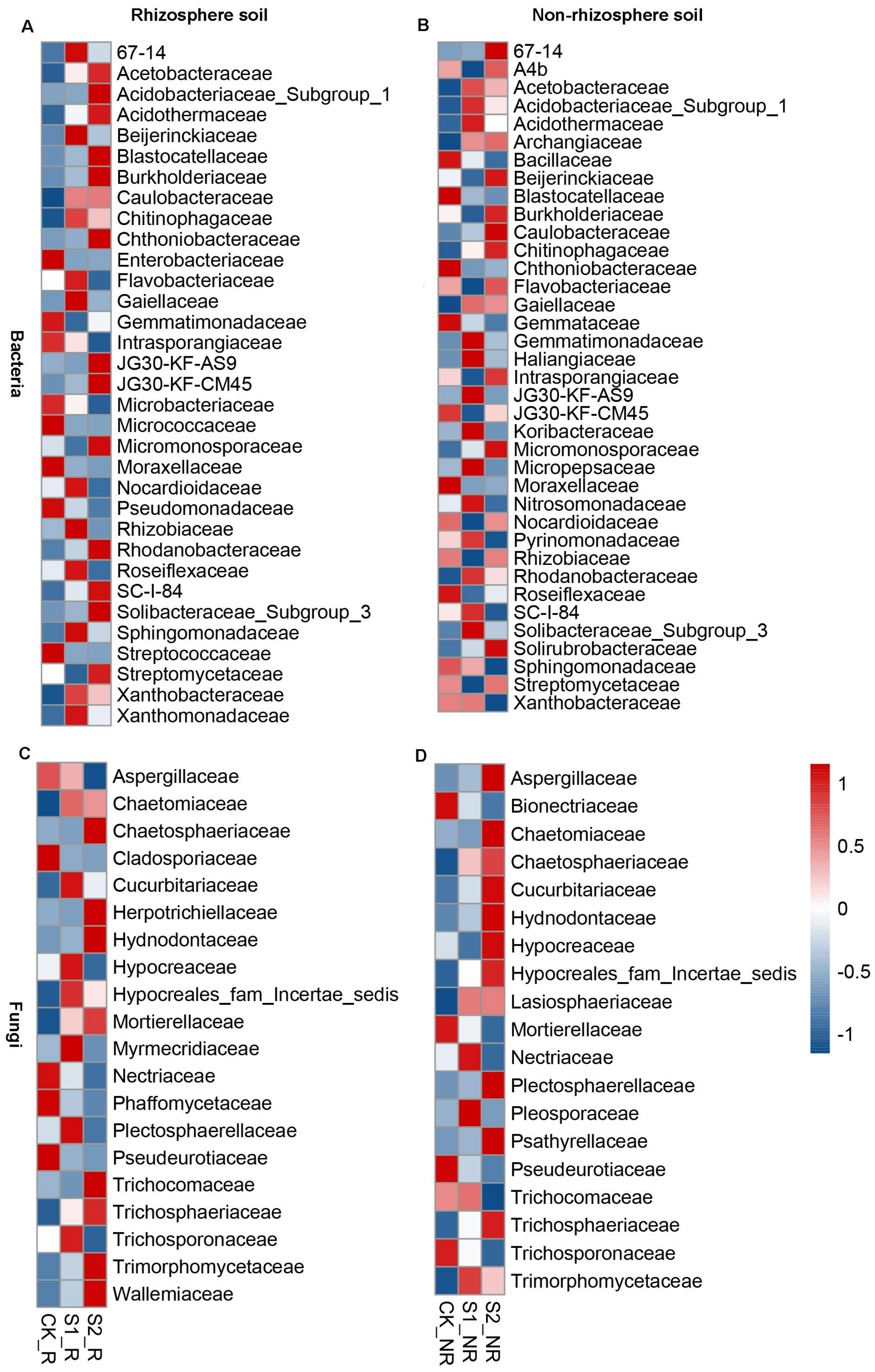

Fig. S2 The microbial community's relative abundance heatmap analysis on the family level (relative abundance ≥ 1 % was chosen). A) the bacterial community in the maize rhizosphere; **B**) the bacterial community in the maize non-rhizosphere; **C**) the fungal community in the maize rhizosphere; **D**) the fungal community in the maize non-rhizosphere.

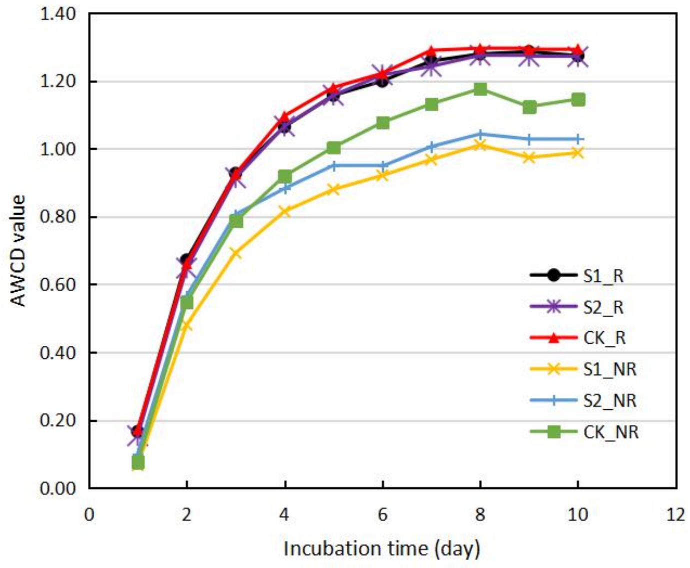

Fig. S3 The dynamic change of the AWCD value over the incubation time.

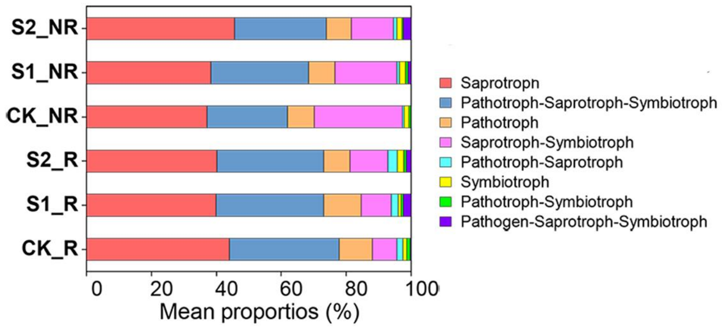

Fig. S4 The function annotation of fungal trophic modes based on FUNGuild with straw mulch in maize rhizosphere and non-rhizosphere soil.

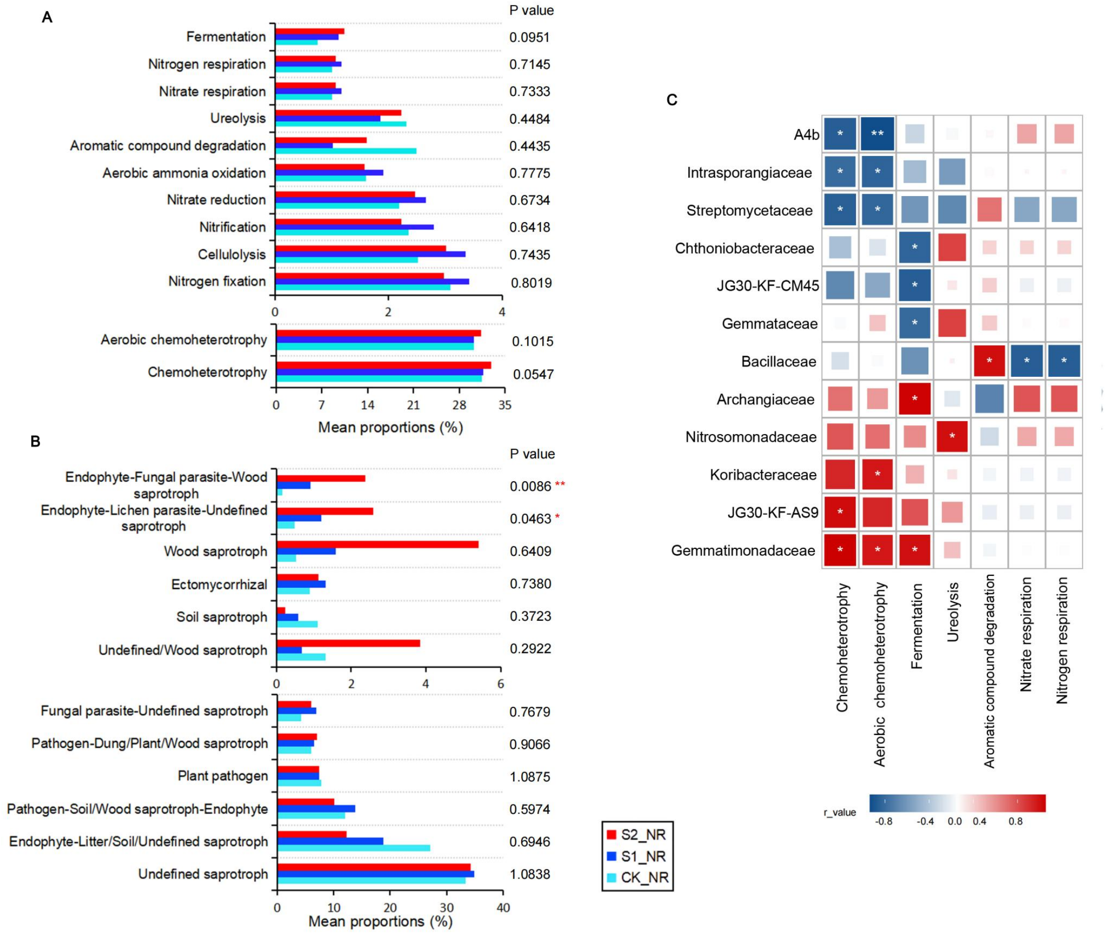

Fig. S5 The functional predictions of the microbial community with straw mulch in maize non-rhizosphere soil. A) Bacterial function annotation by FAPROTAX with straw mulch in maize non-rhizosphere soil (the mean proportions > 1%); **B**) Function annotation of a fungal guild type based on FUNGuild with straw mulch in maize non-rhizosphere soil (the mean proportions of > 1% were shown). C) The correlation heatmap graph between bacterial environment functions and species (family level, relative abundance > 1%) in maize non-rhizosphere soil with straw mulch. "*", "**", and "***" represent significant differences at $P < 0.05$, $0.01 < P < 0.05$, $0.001 < P < 0.01$ level, respectively. Use the Benjamini and Hochberg method to correct P values.

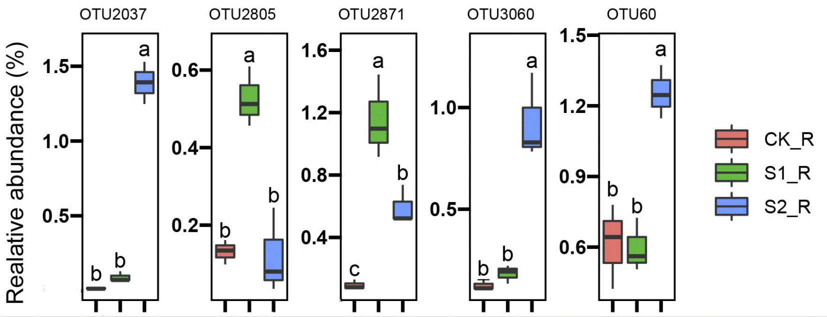

OTUs	Taxonomy				Trophic type	
	Phylum	Class	Order	Family	Trophic Mode	Guild
OTU2805	Ascomycota	Sordariomycetes	Sordariales	Chaetomiaceae	Saprotroph	Undefined/Wood Saprotroph
OTU3060	Ascomycota	Sordariomycetes	Sordariales	Chaetomiaceae	Saprotroph	Undefined/Wood Saprotroph
OTU60	Ascomycota	Sordariomycetes	Chaetosphaeriales	Chaetosphaeriaceae	Symbiotroph	Ectomycorrhizal
OTU2037	Ascomycota	Sordariomycetes	Chaetosphaeriales	Chaetosphaeriaceae	Saprotroph-Symbiotroph	Endophyte-Litter/Wood Saprotroph
OTU2871	Ascomycota	Sordariomycetes	Hypocreales	Hypocreales_fam_Incertae_sedis	Pathogen-Saprotroph-Symbiotroph	Animal/Plant Pathogen-Endophyte-Fungal Parasite-Wood Saprotroph

Fig. S6 The main fungal species involved significant differences in guild types in maize rhizosphere soil with

straw mulch. The different lower cases mean significant differences among treatments.

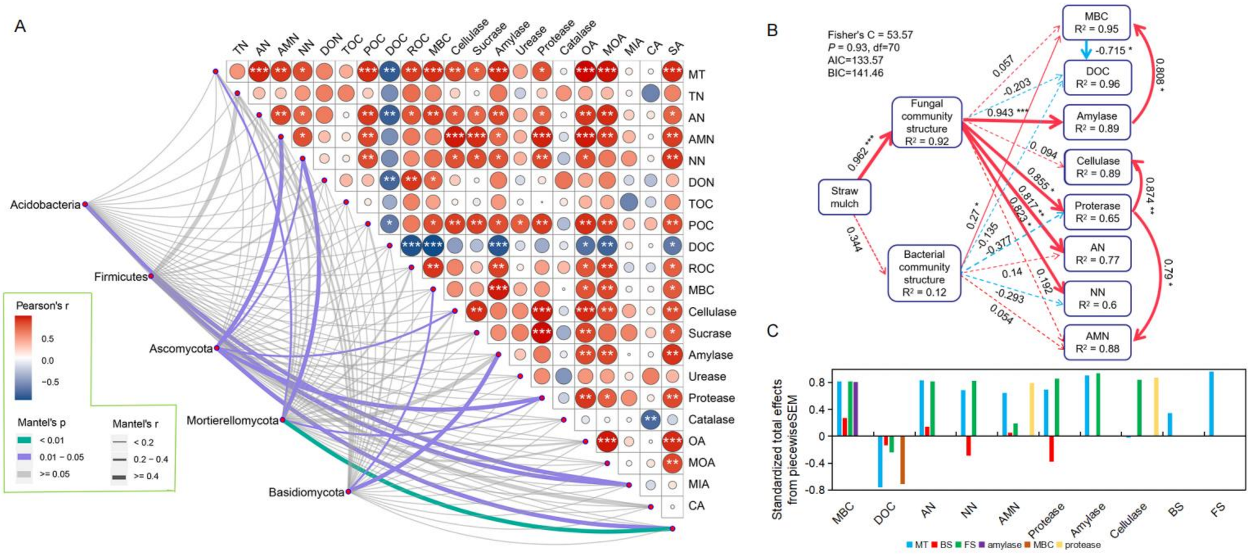

Fig. S7 The relationship between the microbial community and soil factors in maize non-rhizosphere soil with straw mulch. A) The correlation between microbial community (phylum level) and soil factors in maize non-rhizosphere soil with straw mulch. The right-upper shows the Pearson correlation among components of soil carbon and nitrogen, enzyme activity, and organic acids. The left-bottom shows the Mantel analysis between the microbial community and soil factors. The width and color of the lines show different Mantel's r and p values. Just present the microbial community that has a significant correlation. **B**) The piecewise structural equation models (SEMs) show the direct and indirect correlation among straw mulch, microbial community structure (Bray_curtis distance), and soil factors in maize non-rhizosphere soil with straw mulch. Solid and dashed lines with arrows denote significant and non-significant correlations, respectively. Red and blue lines with an arrow indicate positive and negative effects, respectively. The width of lines with an arrow shows the standardized path coefficients, and the special values are labeled on the adjacent lines. The value of R^2 in boxes denotes the response explained variance. **C**) The standardized total effects among straw mulch, bacterial community structure, and soil factors are calculated with piecewise structure equation models. MT: straw mulch treatments; TN: total nitrogen; AN: available nitrogen; AMN: ammonium nitrogen; NN: nitrate nitrogen; DOC: dissolved organic nitrogen; TOC: total organic carbon; POC: particulate organic nitrogen; ROC: readily-oxidizable organic carbon; MBC: microbial biomass carbon; OA: oxalic acid; MOA: malonic acid; MIA: malic acid; CA: citric acid; SA: succinic acid. Significant difference levels: *** $P < 0.001$; ** $P < 0.01$; * $P < 0.05$.
